# Access to affordable medicines and diagnostic tests for asthma and COPD in sub Saharan Africa: the Ugandan perspective

**DOI:** 10.1186/s12890-017-0527-y

**Published:** 2017-12-08

**Authors:** Davis Kibirige, Leaticia Kampiire, David Atuhe, Raymond Mwebaze, Winceslaus Katagira, Winters Muttamba, Rebecca Nantanda, William Worodria, Bruce Kirenga

**Affiliations:** 1 Department of Medicine, Uganda Martyrs Hospital Lubaga, P.O. BOX 7146, Kampala, Uganda; 2 Medical unit, GlaxoSmithKline Pharmaceutical Kenya Limited, Kampala, Uganda; 3 Infectious Disease Research Collaboration (IDRC), Kampala, Uganda; 4 Department of Medicine, Case hospital Kampala, Kampala, Uganda; 5 Department of Medicine, St. Francis hospital Nsambya, Kampala, Uganda; 6 Makerere Lung Institute, Makerere University College of Health Sciences, Kampala, Uganda; 7 Department of Paediatrics and Child Health, Makerere University College of Health Sciences, Kampala, Uganda; 8 Division of Pulmonology, Mulago National Referral and Teaching hospital, Kampala, Uganda

**Keywords:** Access, Medicines, Diagnostic tests, Asthma, COPD, Sub Saharan Africa, Uganda

## Abstract

**Background:**

Equitable access to affordable medicines and diagnostic tests is an integral component of optimal clinical care of patients with asthma and chronic obstructive pulmonary disease (COPD). In Uganda, we lack contemporary data about the availability, cost and affordability of medicines and diagnostic tests essential in asthma and COPD management.

**Methods:**

Data on the availability, cost and affordability of 17 medicines and 2 diagnostic tests essential in asthma and COPD management were collected from 22 public hospitals, 23 private and 85 private pharmacies. The percentage of the available medicines and diagnostic tests, the median retail price of the lowest priced generic brand and affordability in terms of the number of days’ wages it would cost the least paid public servant were analysed.

**Results:**

The availability of inhaled short acting beta agonists (SABA), oral leukotriene receptor antagonists (LTRA), inhaled LABA-ICS combinations and inhaled corticosteroids (ICS) in all the study sites was 75%, 60.8%, 46.9% and 45.4% respectively. None of the study sites had inhaled long acting anti muscarinic agents (LAMA) and inhaled long acting beta agonist (LABA)-LAMA combinations. Spirometry and peak flow-metry as diagnostic tests were available in 24.4% and 6.7% of the study sites respectively. Affordability ranged from 2.2 days’ wages for inhaled salbutamol to 17.1 days’ wages for formoterol/budesonide inhalers and 27.8 days’ wages for spirometry.

**Conclusion:**

Medicines and diagnostic tests essential in asthma and COPD care are not widely available in Uganda and remain largely unaffordable. Strategies to improve access to affordable asthma and COPD medicines and diagnostic tests should be implemented in Uganda.

## Background

Globally, chronic respiratory diseases pose a major public health threat. Notably, the burden of asthma and chronic obstructive pulmonary disease (COPD) is steadily increasing in both developed and developing countries. According to recent World Health Organisation (WHO) estimates, about 235 million people have asthma and 65 million people have moderate to severe COPD. High rates of mortality due to both conditions have been documented in low-and middle income countries (LMIC). In 2012, > three million people died of COPD, which accounted for about 6% of the all the deaths globally. An estimated 90% of these deaths occurred in LMIC [1].

In Uganda, a similar growing trend of mortality related to asthma and COPD has been described. A descriptive retrospective study conducted at an urban national referral hospital reported the burden of asthma and COPD of 70.6% and 21.6% respectively in 558 patients admitted to the hospital’s adult pulmonology unit between December 2010 and August 2011. In hospital mortality among the patients admitted with asthma and COPD was 8.3% and 9.3% respectively [2]. Another population based prospective cross sectional study performed in rural Masindi, a district in Western Uganda in 2012 reported the burden of COPD of 16.2% especially among participants of both gender aged 30–39 years [3]. This highlights that the COPD in Uganda primarily occurs among the young and this could probably be due to effects of environmental factors like increased use of biomass fuel for cooking and early infections like tuberculosis and recurrent childhood pneumonia. The first and recently concluded nationwide population survey to determine the burden and predictors of asthma in Uganda, the Uganda National Asthma Survey (UNAS) has documented a prevalence of 9.8% with a higher prevalence of asthma reported in urban areas compared to rural areas (Kirenga B et al. Unpublished data, 2016).

Despite the increasing burden of asthma and COPD and related high mortality rates in LMIC, suboptimal care of patients with asthma and COPD is still very common in LMIC. The challenges of low availability of affordable medicines and diagnostic tests for asthma and COPD, poor health system structures coupled with low knowledge levels of asthma and COPD management among healthcare workers and patients contribute to the suboptimal care in LMIC [4–8].

The WHO strategy on secondary and tertiary prevention section of chronic respiratory diseases emphasises the need to strengthen health care by identifying cost-effective interventions, upgrading standards and accessibility of care at different levels of the health care system and improving access to affordable medicines [9]. Improving access to affordable medicine is one of the key components of the millennium development goals (MDG) referred to as the MDG target 8E [10].

The main objective of this study was to provide contemporary information about the availability, cost and affordability of selected essential medicines and diagnostic tests for asthma and COPD in Uganda, as recommended by the Global INitiative for Asthma (GINA) guidelines [11], Global initiative for chronic Obstructive Lung Disease (GOLD) guidelines [12], WHO guidelines of asthma and COPD management and are included in the WHO essential drug list for management of asthma and COPD [1, 13] and Uganda Clinical Guidelines (UCG).

This data will help guide a pragmatic approach to address the low access to affordable asthma and COPD medicines and diagnostic tests. It will also guide healthcare managers in Uganda on resource mobilisation and allocation for essential medicines and diagnostic tests for asthma and COPD. The data obtained will add to the existing literature on extent of availability, cost and affordability of essential asthma and COPD medicines and diagnostic tests in LMIC.

## Methods

### Study settings and selection of study sites

**Fig. 1:**
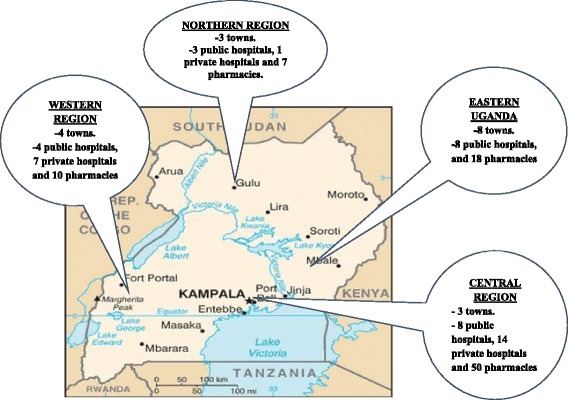
Geographical location of the selected study sites. Map of Uganda obtained from Wikimedia Commons. https://commons.wikimedia.org/wiki/Maps_of_Uganda#/media/File:Ug-map.png

This was a cross sectional study that was conducted from 15th January 2017 to 28th February 2017 in 23 public hospitals, 22 private hospitals and 85 privately owned pharmacies that were randomly selected from the 4 regions of Uganda. The central, eastern, western and northern regions accounted for 55.4%, 20%, 16.2% and 8.4% of the selected study sites respectively (Fig. 1 shows geographical location of the study sites).

All the selected hospitals were tertiary hospitals, served a significantly large local population in each geographical location and had a daily or weekly functioning outpatient general medical or respiratory clinic. None was a specialist hospital. The pharmacies selected were those in close proximity to a tertiary hospital or a health centre IV (secondary hospital) serving a large local population and are licensed by the regulatory body (National Drug Authority) to sell all classes of medicines.

A total of 2 public and 2 private hospitals were excluded because they lacked a regular outpatient medical specialist clinic. Drug shops were not included because they are not licensed to sell essential medicines for NCD. A total of 30 pharmacies were excluded because the staff declined to provide the desired information on availability and cost of the study drugs.

According to the registry of ministry of Health, Republic of Uganda, there are 2 national referral hospitals, 14 regional referral hospitals and 139 general hospitals. About 41.9% are government owned, 40.7% are private not for profit (PNFP) and 17.4% are private for profit [14].

Medical care in the public hospitals is paid for the government of Uganda. A national procurement institution called the National Medical Stores (NMS) is constitutionally mandated to procure and distribute all drugs and diagnostic tests for all government hospitals. Despite this, stock out of drugs and diagnostic tests is a common finding in most public hospitals. Patients seek medical care from private hospitals and pharmacies as a second option in cases of unavailability of drugs and diagnostic tests. These procure their drugs and diagnostic tests from private companies. According to 2016/17 Uganda National Household Survey (UNHS) conducted by the Uganda Bureau of Statistics (UBOS) to collect information on socio-economic characteristics at both household and community levels with an aim of monitoring development performance of key sectors, about 48% and 34% of surveyed participants sought medical care from private clinics/hospitals and public hospitals respectively. Patients also sought medical care from private pharmacies and village health workers [15].

About 599 privately owned retail pharmacies and 90 private hospital pharmacies that sell human pharmaceutical drugs are registered by the National Drug Authority. About more than 70% of these are in the central region [16].

This study was conducted as a sub-section of the large study called the Access to Cardiovascular diseases, Chronic Obstructive pulmonary disease, Diabetes mellitus and Asthma Drugs and diagnostics (ACCODAD) study that assessed the availability and affordability of essential medicines and diagnostic tests of non-communicable diseases (NCD) in Uganda. The first published study from the ACCODAD study assessed access to key drugs and diagnostic tests in the management of diabetes and cardiovascular diseases [17].

### Sample size estimation

Basing on one of the primary objectives of the ACCODAD study which was to determine the extent of availability of the medicines and diagnostic tests of interest, the overall availability of Pulmicort® (budesonide) in a study by Babar Z et al. in 52 surveyed LMICs of 8.3% was used as the prevalence (P) [7]. Using the formula: *n* = Z2 P (1-P)/d2 where Z (normal value corresponding to the 95% confidence interval) =1.96, *P* = 0.083 and d = 0.05 (desired precision of estimation), a sample size of 117 healthcare units (public and private hospitals and private pharmacies) was obtained. We increased the sample size of the surveyed health units to 130 to generate adequate information. We aimed at surveying a minimum of 10% of the registered public and private hospitals and private pharmacies in Uganda.

### Data collection

Data was collected using a pre tested questionnaire based on the WHO and Health Action International (HAI) standardised methods of assessing medicine availability, prices and affordability in LMIC [18] from 15th January 2017 to 28th February 2017. The study data collection team underwent a brief training before commencement of the study to improve quality and standardisation of data.

The diagnostic tests of interest of asthma and COPD in this study were spirometry and peak flow-metry. The medicines of interest were: inhaled short acting beta agonists (SABA) like salbutamol meter dose inhaler (MDI) 100 μg, inhaled short acting anti muscarinic agents (SAMA) like ipratropium bromide 20 and 40 μg MDI, inhaled long acting anti muscarinic agents (LAMA) like tiotropium 18 μg dry powder inhalers (DPI), inhaled SABA and SAMA combinations like salbutamol/ipratropium 100/20 μg soft mist inhaler (SMI), inhaled LABA and LAMA combinations like olodaterol/tiotropium 5/5 μg SMI, inhaled LABA-inhaled corticosteroid (ICS) combinations like formoterol-budesonide 4.5/160 μg MDI and salmeterol-fluticasone propionate 25/125 μg MDI, ICS like budesonide 200 μg DPI and fluticasone propionate 250 μg DPI, oral methylxanthines like slow release theophylline 100 mg and oral leukotriene receptor antagonists (LTRA) like tablets montelukast 10 mg and 20 mg. Adult and paediatric spacer devices were also included due to their integral role in asthma and COPD management especially in young children and elderly patients.

Information about the availability of each stated medicine category and monthly cost of the available lowest priced generic medicine was obtained. The cost of the medicines obtained was the retail prices charged directly to the patients at the respective pharmacies of the private hospitals and private pharmacies. Information about the availability and cost of the diagnostic tests of interest was only obtained from the hospitals.

The cost of the medicines in the public hospitals was not obtained since medical care is offered free of charge. All the costs of the medicines and the diagnostic tests were obtained in Uganda shillings (UgX) and converted to US dollars (USD) according to the existing exchange rate at the time of data collection (1 USD = 3600 UgX).

### Data analysis

Availability of the medicine categories were assessed using simple descriptive statistics by calculating the proportions of hospitals and private pharmacies in which any type and dose of the medicine and diagnostic test was present on the day of data collection at the study site. The study definition of low, moderate and high availability of the selected medicines and diagnostic tests at any study site was <50%, 50–79% and ≥80% respectively. The availability of the selected diagnostic tests and medicines was compared between the study sites and study regions using chi-square test to determine significant differences. A *p* value of <0.05 was considered as statistically significant.

The unit prices of the medicines in USD were converted to a median price ratio (MPR) by dividing the median local price by an international reference price (IRP). The IRP is obtained from the Management Sciences for Health International Drug Price Indicator Guide which reports median prices of high quality multisource medicines offered to LMICs countries by different suppliers. The MPR is used to express how much greater or less the median local medicine price is than the IRP. An MPR of 3 would mean that the local medicine price is three times greater than the IRP. The study definition of reasonable pricing was when the MPR of a patient’s medicine was <1.5 [19]. The cost of the available lowest priced generic medicine was compared to the cost of the available originator brand medicine.

Affordability was estimated using the median monthly medicine prices and the average salary of the lowest paid government worker in USD and calculating the number of days’ wages required to purchase a one-month course of treatment or to pay for a specific diagnostic test. Medicines and diagnostic tests that cost ≤3 days’ wages were considered affordable.

The lowest paid skilled government worker at the time of the study (scale U8 lower-non formal education teachers) earns a gross salary of 198,793 UgX equivalent to 55.2 USD. After tax deductions, this translates to a net salary of 139,155 UgX equivalent to 38.7 USD per month or 4638.5 UgX (1.3 USD daily) [20].

## Results

### Availability of the medicines and diagnostic tests

The availability of the selected medicines ranged from 0% for inhaled LAMA and inhaled LAMA-LABA combinations to 75% for SABA. Inhaled SABA-SAMA combinations, inhaled SAMA, oral methylxanthines, ICS, LABA-ICS and oral LTRA were available in 10.8%, 12.3%, 16.9%, 45.4%, 46.9% and 60.8% respectively in all study sites. Adult and paediatric spacers were available in only 18.5% and 19.2% of study sites respectively. Spirometry and peak flow-metry were available in only 24.4% and 6.7% of the study sites respectively (summarised in Table 1).

**Table 1:** Availability of the asthma-COPD drugs and diagnostic tests in private and public hospitals and private pharmacies (*n* = 130)

		Availability (%)			
A: Selected medicines (*N* = 10 classes and 2 types of spacers)	Availability (%) in all study sites (*N* = 130)	Public hospitals (*N* = 23)	Private hospitals (*N* = 22)	Private pharmacies (*N* = 85)	*P* value
Inhaled LAMA monotherapies	0.0	0.0	0.0	0.0	NA
Inhaled LABA and LAMA combinations	0.0	0.0	0.0	0.0	NA
Inhaled LABA monotherapies	10.0	0.0	0.0	15.3	0.022
Inhaled SABA and SAMA combinations.	10.8	0.0	4.6	15.3	0.065
Inhaled SAMA monotherapy	12.3	0.0	0.0	18.8	0.008
Oral methylxanthines	16.9	4.0	18.2	20.0	0.203
Adult spacer devices	18.5	0.0	0.0	28.2	<0.001
Paediatric spacer devices	19.2	0.0	4.6	28.2	0.002
ICS monotherapies	45.4	4.0	50.0	55.3	<0.001
Inhaled LABA- ICS combinations	46.9	0.0	40.9	61.2	<0.001
Oral LTRA	60.8	0.0	59.1	76.7	<0.001
Inhaled SABA monotherapy	75.0	26.1	77.3	88.2	<0.001
B: Diagnostic/monitoring tests					
Peak flow-metry	6.7	8.7	4.6	NA	0.577
Spirometry	24.4	34.8	13.6	NA	0.099

LAMA-Long acting anti muscarinic agents, LABA-Long acting beta agonists, SABA-Short acting beta agonists, SAMA-Short acting anti muscarinic agents, ICS-Inhaled corticosteroids, LTRA-Leukotriene receptor antagonists

### Comparison of the availability of selected medicines in the study sites and regions

There was a stark difference in availability of medicines in the study sites and regions. Low availability of medicines was mostly documented in the public hospitals compared to private hospitals and pharmacies. The only available medicines in the public hospitals were: inhaled SABA in 26.1% compared to 77.3% and 88.2% in private hospitals and pharmacies respectively (*p* < 0.001), ICS in 4.4% compared to 50% and 55.3% in private hospitals and pharmacies respectively (p < 0.001) and oral methylxanthines in 4.4% compared to 18.2% and 20% in private hospitals and pharmacies respectively (*p* = 0.203). Comparing private hospitals and private pharmacies, the selected medicines were more available in the latter. Of the 3 study sites categories, inhaled LABA, SAMA and adult spacers were only available in private pharmacies (15.3%, 18.8% and 28.2% respectively) (summarised in Table 1). All the medicines of interest except inhaled LABA were more available in the central region (summarised in Table 2).

**Table 2:** Availability of the asthma-COPD drugs in the 4 study regions

	Availability (%)				
Selected medicines (*N* = 10 classes and 2 types of spacers)	Central region	Eastern region	Western region	Northern region	*P* value
Inhaled LABA and LAAC combinations	0.0	0.0	0.0	0.0	NA
Inhaled LAAC monotherapies	0.0	0.0	0.0	0.0	NA
Inhaled LABA monotherapies	9.7	4.2	4.6	33.3	0.031
Inhaled SABA and SAAC combinations	19.4	0.0	0.0	0.0	0.005
Inhaled SAAC monotherapy	22.2	0.0	0.0	0.0	0.002
Oral methylxanthines	22.2	16.7	4.6	8.3	0.215
Adult spacer devices	25.0	8.3	13.6	8.3	0.184
Paediatric spacer devices	30.6	4.2	4.6	8.3	0.004
Inhaled LABA-ICS combinations	61.1	25.0	31.8	33.3	0.004
ICS monotherapies	61.1	37.5	9.1	33.3	<0.001
Oral LTRA	75.0	37.5	50.0	41.7	0.002
Inhaled SABA monotherapy	80.6	70.8	68.2	66.7	0.490

LAMA-Long acting anti muscarinic agents, LABA-Long acting beta agonists, SABA-Short acting beta agonists, SAMA-Short acting anti muscarinic agents, ICS-Inhaled corticosteroids, LTRA-Leukotriene receptor antagonists

### Comparison of the availability of selected diagnostic tests in private and public hospitals

There was no documented statistically significant difference in the availability of spirometry and peak flow-metry in private and public hospitals. Spirometry was available in 13.6% and 34.8% of private and public hospitals respectively (*p* = 0.099) while peak flow-metry were available in 4.6% and 8.7% of private and public hospitals respectively (*p* = 0.577) (summarised in Table 1).

### Comparison of the median cost of the lowest priced generic and originator brand in the private hospitals and pharmacies

With the exception of Combivent® (salbutamol/ipratropium 100/20 μg soft mist inhaler), the median prices of all originator brands were higher than the obtained median prices of the generic brands. Marked differences in median prices were noted with formoterol/budesonide 4.5/160 μg LABA-ICS combination, ICS budesonide 200 μg and all doses of the ICS fluticasone propionate 250 μg, 125 μg and 50 μg. The cost of generic and originator montelukast 20 mg brands were similar (summarised in Table 3).

**Table 3:** Comparison of the median prices of the inhaled and oral generic and originator asthma and COPD medicine brands

Generic brands	Median (IQR) price in UgX	Median (IQR) price in USD	Originator brands	Median (IQR) price in UgX	Median (IQR) price in USD
Salbutamol 100 μg	10,000 (9000–13,000)	2.8 (2.5–3.6)	Ventolin 100 μg	12,000 (10000–15,000)	3.3 (2.8–4.2)
Ipratropium bromide 20 μg	60,000 (48500–65,000)	16.7 (13.5–18.1)	Atrovent 20 μg	62,500 (60000–65,000)	17.4 (16.7–18.1)
Ipratropium 40 μg	64,000 (56750–65,000)	17.8 (15.8–18.1)	Atrovent 40 μg	65,000 (65000–70,000)	18.1 (18.1–19.4)
Salbutamol/ipratropium 100/20 μg	50,000 (35000–65,000)	13.9 (9.7–18.1)	Combivent 100/20 μg	40,000 (30000–50,000)	11.1 (8.3–13.9)
Formoterol-budesonide 4.5/160 μg	80,000 (45000–85,000)	22.2 (12.5–23.6)	Symbicort 4.5/160 μg	100,000 (85000–120,000)	27.8 (23.6–33.3)
Salmeterol-fluticasone propionate 25/125 μg	48,000 (35000–50,000)	13.3 (9.7–13.9)	Seretide 25/125 μg	50,000 (48000–60,000)	13.9 (13.3–16.7)
Budesonide 200 μg	37,500 (33000–42,000)	10.4 (9.2–11.7)	Pulmicort 200 μg	100,000 (75000–130,000)	27.8 (20.8–36.1)
Fluticasone propionate 250 μg	25,000 (25000–26,000)	6.9 (6.9–7.2)	Flixotide 250 μg	87,500 (80000–95,000)	24.3 (22.2–26.4)
Fluticasone propionate 125 μg	27,000 (25000–40,000)	7.5 (6.9–11.1)	Flixotide 125 μg	87,500 (87500–87,500)	24.3 (24.3–24.3)
Fluticasone propionate 50 μg	27,500 (25000–30,000)	7.6 (6.9–8.3)	Flixotide 50 μg)	78,500 (78500–78,500)	21.8 (21.8–21.8)
Tablets montelukast 10 mg	1000 (800–1200)	0.3 (0.2–0.3)	Singular 10 mg	1100 (900–1400)	0.3 (0.3–0.4)
Tablets montelukast 20 mg	1200 (1000–1500)	0.3 (0.3–0.4)	Singular 20 mg	1200 (1100–1500)	0.3 (0.3–0.4)

IQR-Inter-quartile range, UgX-Uganda shillings, USD-US dollars

### Pricing and affordability of the medicines and diagnostic tests

#### Selected medicines

The IPR were only available for salbutamol 100 μg inhalers, ipratropium 20 μg inhalers, beclometasone 100 μg inhalers, budesonide 200 μg inhalers and fluticasone propionate 125 μg. According to the calculated MPR, these drugs were all highly priced with the local retail prices being >100 times the IRP.

Affordability of the drugs ranged from 2 days’ wages for salbutamol 100 μg metered dose inhalers to 17 days’ wages for formoterol-budesonide 4.5/160 μg LABA-ICS combination. According to the study definition of affordability, salbutamol 100 μg MDI was the only affordable asthma and COPD medicine. With the exception of fluticasone propionate 125 μg inhalers (cost 10 days’ wages) and budesonide 200 μg inhalers (cost 8 days’ wages), the rest of controller or maintenance medicines cost ≤7 days’ wages.

Inhaled ipratropium either as monotherapy or in combination with salbutamol as reliever medicines were largely unaffordable, with costs of up to 8 to 14 days’ wages. Both adult and paediatric spacers were also largely unaffordable (costs of 13 and 8 days’ wages respectively).

(Pricing and affordability of medicines is summarised in Table 4).

**Table 4:** Median (IQR) prices and affordability of the lowest priced generic inhaled asthma-COPD drugs in both private hospitals and pharmacies

Drug	Median (IQR) price in Ug Shs	Median local price in USD	IRP in USD	MPR	Monthly cost in USD	Days’ wages
Salbutamol 100 μg	10,000 (9000–13,000)	2.8	0.0114	243	2.8	2.2
Formoterol 12 μg.	38,500 (30000–44,000)	10.7	–	–	10.7	8.2
Salmeterol 25 μg	30,000 (25500–33,750)	8.3	–	–	8.3	6.4
Ipratropium bromide 20 μg	60,000 (48500–65,000)	10.7	0.0220	486	10.7	8.2
Ipratropium 40 μg	64,000 (56750–65,000)	17.8	–	–	17.8	13.7
Salbutamol/ipratropium 100/20 μg	50,000 (35000–65,000)	13.9	–	–	13.9	10.7
Formoterol-beclomethasone 6/100 μg	30,000 (15000–40,000)	8.3	–	–	8.3	6.4
Formoterol-budesonide 4.5/160 μg	80,000 (45000–85,000)	22.2	–	–	22.2	17.1
Salmeterol-fluticasone propionate 25/125 μg	48,000 (35000–50,000)	13.3	–	–	13.3	10.2
Beclomethasone dipropionate 100 μg	25,000 (20000–30,000)	6.9	0.0444	155	6.9	5.3
Budesonide 200 μg	37,500 (33000–42,000)	10.4	0.0305	340	10.4	8
Fluticasone propionate 250 μg	25,000 (25000–26,000)	6.9	–	–	6.9	5.3
Fluticasone propionate 125 μg	27,000 (25000–40,000)	7.5	0.0630	119	7.5	5.8
Fluticasone propionate 50 μg	27,500 (25000–30,000)	7.6	–	–	7.6	5.9
Slow release theophylline tablets 100 mg	1000 (1000–1900)	0.3	–	–	9	6.9
Tablets montelukast 10 mg	1000 (800–1200)	0.3	–	–	9	6.9
Tablets montelukast 20 mg	1200 (1000–1500)	0.3	–	–	9	6.9
Adult spacers	60,000 (51000–65,000)	16.7	–	–	16.7	12.9
Paediatric spacers	35,000 (28500–45,000)	9.7	–	–	9.7	7.5

IQR-Inter-quartile range, UgX-Uganda shillings, USD-US dollars, IRP-International reference price, MPR-Median price ratio

#### Diagnostic tests

The cost of performing spirometry was obtained from only 2 surveyed hospitals because the other hospitals offer it free of charge. The median cost of performing spirometry was 130,000 UgX (36.1 USD) which translates to 27.8 days’ wages.

## Discussion

### Availability of the medicines and diagnostic tests

This study aimed at providing contemporary information about the availability, cost and affordability of medicines and diagnostic tests essential in asthma and COPD management in Uganda. To our knowledge, this is the largest study to investigate the availability, cost and affordability of these essential medicines and diagnostic tests in Uganda.

Generally, all the selected asthma and COPD medicines and diagnostic tests were either of moderate or low availability. The lowest levels of availability were in public hospitals. The only available medicines in the public hospitals were inhaled SABA in 26.1%, ICS and oral methylxanthines (both in 4.4%). This low availability of essential medicines of asthma and COPD in our study has also been widely reported in the majority of studies investigating access to chronic diseases medicines in LMIC [7, 21–23] and in the annual medicine price monitor studies in Uganda [24, 25].

In the study by Babar Z et al. performed in 52 LMIC (Uganda inclusive) to investigate the availability, pricing and affordability of 3 essential asthma medicines (inhaled salbutamol, beclometasone and budesonide); generic beclometasone and budesonide were available in only 19% and 16% of the surveyed public hospitals respectively and in 46% and 58% in the private sector respectively. The 2 ICS of interest were unavailable in 14 of the surveyed 52 LMIC. Generic salbutamol inhalers were available in 56% and 82% of the public hospitals and private pharmacies [7]. Similar findings of low availability of asthma medicines was also reported by another cross sectional study by Cameron A et al. performed in 36 LMIC (10 sub Saharan African countries including Uganda). This study sought to assess the availability and pricing of 30 core medicines for acute and chronic diseases. The only surveyed asthma drug (inhaled salbutamol 100 μg MDI) was on average available in 14% (0–55.9%) and 47% (0–95%) of the public hospitals and private pharmacies in the 8 participating African countries [21].

According to the medicine price monitoring study conducted in Uganda in 2014, the availability of the only assessed asthma medicine (salbutamol 100 μg inhaler) was 13%, 54% and 56% in the surveyed public, private and mission or PNFP health facilities [22]. The 2015 medicine price monitoring study noted that salbutamol inhaler was available in <25% of the public facilities [23].

The dismal availability of asthma and COPD medicines in Uganda especially in the public hospitals could be due to the few asthma and COPD medicines included in our essential drug list. Among the medicines of interest for this study, the asthma medicines section of the 2012 Uganda essential drug list included only aminophylline tablets, beclometasone and salbutamol inhalers [26]. These are also the only drugs recommended for management of asthma in the 2016 Uganda clinical guidelines (UCG). There is no recommendation for inhaled LABA-ICS combinations, inhaled LAMA, inhaled SAMA and oral LTRA in the management of asthma. Despite the 2016 UCG recommendation to use ipratropium inhalers and inhaled LABA in combination with ICS in the management of COPD, both drugs were absent in all surveyed public and private hospitals. Ipratropium, a key SAMA was only available in private pharmacies (18.8%).

Other essential medicines in COPD management like inhaled LAMA (either as monotherapy/in combination with LABA) were not available in any study site. This could also probably be due to their absence in the Ugandan clinical guidelines and essential drug lists. The overall low and moderate availability of the key asthma and COPD medicines in the study could also be explained by the evident knowledge gaps and inapt prescription practices among healthcare practitioners in Uganda with regard to asthma and COPD management. A retrospective study evaluating asthma medication prescription practices of healthcare practitioners at the chest clinic and accident and emergency department of a national referral hospital in Uganda noted a high frequency of prescription of oral salbutamol (> 50%), oral prednisolone (64.2%) and oral aminophylline (16%) in the chest clinic. Inhaled beta agonists and ICS were only used in 38% and 24% of the patients [5].

Low availability of spacers which are key in facilitating optimal delivery of inhaled medicines in young children and elderly patients with poor inspiratory effort (<20% in all study sites) and the diagnostic tests (spirometry and peak flow-metry <25% and <10% respectively) was well documented in both the public and private sector. Availability of spacers and diagnostic tests has not been widely studied in most studies assessing access to asthma and COPD medicines and diagnostics in LMIC.

### Affordability of the medicines and diagnostic tests

With the exception of salbutamol inhalers, the rest of the asthma and COPD medicines were largely unaffordable when accessed from the private sector. From the list of medicines of interest, the cheapest inhaled LABA (salmeterol 25 μg MDI), inhaled SAMA (ipratropium 20 μg), inhaled SABA-SAMA combination (salbutamol/ipratropium 100/20 μg), ICS (beclometasone 100 μg /fluticasone propionate 250 μg) and inhaled LABA-ICS combination (formoterol-beclometasone 6/100 μg) would cost 6 days’ wages, 8 days’ wages, 11 days’ wages, 5 days’ wages and 6 days’ wages of the least paid government employee respectively. All recommended oral controller therapies (methylxanthines and LTRA) would cost about 7 days’ wages.

The cheapest standard generic asthma and COPD treatment regimen of an inhaled SABA (used on an as needed basis) and ICS-LABA combination would collectively cost 11.1 USD which translates to 8.6 days’ wages of the least paid government employee. A combination regimen of the cheapest generic SABA inhaler (salbutamol inhaler 100 μg) and ICS (beclometasone inhaler 100 μg) would cost 9.7 USD or 7.5 days’ wages.

A wide variation in affordability of asthma and COPD medicines has been reported in the majority of similar studies across different countries. In one study investigating the availability and affordability of essential medicines in 6 LMIC documented the cost of standard asthma combination therapy of the lowest priced generic salbutamol and beclometasone inhalers were 1.3 days’ wages in Bangladesh, 9.2 days’ wages in Malawi, 5.4 days’ wages in Nepal, 2.5 days’ wages in Pakistan and 2.3 days’ wages in Sri Lanka [22]. The cost of similar combination therapy in another study performed in India was 2 days’ wages [27]. Salbutamol inhaler remains affordable in Uganda. The 2014 and 2015 medicine price monitor studies performed in Uganda reported the cost of only salbutamol 100 μg inhaler of 1.4 and 1.6 days’ wages respectively [24, 25] compared to 2.2 days wages’ in our study. Other asthma and COPD drugs were not studied.

The majority of asthma and COPD medicines being unaffordable in the private sector in this study could probably be explained by the lack of a national policy or legislation to regulate retail prices of medicines especially for chronic diseases. Uganda also lacks a vibrant local pharmaceutical industry sector that would manufacture cheap quality generic asthma and COPD medicines for the public and private sector.

There is paucity of studies investigating the cost and affordability of performing spirometry in LMIC. The cost equivalent to 27.8 days’ wages reported by our study findings is unaffordable to the majority of Ugandan patients with asthma and COPD. This could be due to the limited hospitals and expertise capable of rendering this specialist service.

## Conclusions

This study demonstrated that the majority of asthma and COPD medicines and diagnostic tests were largely unavailable especially in the public hospitals and unaffordable in the private sector. In the public sector, inequity in access to medicines and diagnostic tests for asthma and COPD care can be addressed through increased awareness about optimal management of asthma and COPD among healthcare practitioners and patients, inclusion of other essential asthma and COPD medicines on the national essential drug list and clinical care guidelines, improving forecast accuracy, procurement efficiency and stock handling and boosting local production of cheap quality generic asthma and COPD medicines. At the private sector level, access to affordable medicines can be improved through implementation of national policies aimed at regulating retail prices of medicines especially for chronic care, improving forecast accuracy, procurement efficiency, stock handling and local production of cheap generic medicines. Due to the prohibitive costs of diagnosis and management of asthma and COPD in Uganda, national primary prevention strategies for chronic respiratory diseases should be widely implemented to reduce the disease burden.

### Study limitations

The data was collected at one point in time which does not put into consideration the temporal variations in availability and prices of medicines over time in the different surveyed hospitals and private pharmacies hence, we cannot establish a causal relationship between the investigated outcome of interest and the risk factors. Misreporting (under-or over-reporting) of the data is highly possible due to selection, information or recall bias and the one point in time approach of data collection.

Assessment of drug stock records was not performed to ascertain availability of drugs. We were unable to obtain the national procurement or tender prices of the mandated government institution (NMS) to procure and supply medicines to all government hospitals. These would accurately reflect the prices of the few drugs procured for the public hospitals in comparison the private hospitals and pharmacies. Using the daily wage of the lowest paid unskilled government to calculate affordability of medicines and diagnostic tests has its limitations because a significant proportion of the Ugandan population earns less than 1.3 USD per day.

Despite these limitations, the standardised WHO/HAI methodology that was used has been widely validated. This is the largest study to comprehensively assess the availability and affordability of several locally and internationally guideline recommended inhaled and oral asthma and COPD medicines and diagnostic tests in Uganda.

## Abbreviations

ACCODAD: Access to cardiovascular diseases, chronic obstructive pulmonary diseases, diabetes and asthma drugs and diagnostics study
COPD: Chronic obstructive pulmonary disease
GINA: Global initiative for asthma
GOLD: Global initiative for chronic obstructive lung disease
HAI: Health Action International
ICS: Inhaled corticosteroids
IRP: International reference price
LABA: Long acting beta agonists
LAMA: Long acting anti muscarinic agents
LTRA: Leukotriene receptor antagonists
MPR: Median price ratio
NMS: National medical stores
SABA: Short acting beta agonists
SAMA: Short acting anti muscarinic agents
UgX: Uganda shillings
USD: United States dollars
WHO: World Health Organisation
